# Community-based health insurance programmes and the national health insurance scheme of Nigeria: challenges to uptake and integration

**DOI:** 10.1186/1475-9276-13-20

**Published:** 2014-02-21

**Authors:** Isaac AO Odeyemi

**Affiliations:** 1 Health Economics & Outcomes Research, Astellas Pharma Europe Ltd, 2000 Hillswood Drive, Chertsey KT16 0RS, UK

**Keywords:** Community-Based Health Insurance, CBHI, Healthcare, National Health Insurance Scheme, NHIS, Sub-Saharan Africa, Nigeria

## Abstract

**Background:**

Nigeria has included a regulated community-based health insurance (CBHI) model within its National Health Insurance Scheme (NHIS). Uptake to date has been disappointing, however. The aim of this study is to review the present status of CBHI in SSA in general to highlight the issues that affect its successful integration within the NHIS of Nigeria and more widely in developing countries.

**Methods:**

A literature survey using PubMed and EconLit was carried out to identify and review studies that report factors affecting implementation of CBHI in SSA with a focus on Nigeria.

**Results:**

CBHI schemes with a variety of designs have been introduced across SSA but with generally disappointing results so far. Two exceptions are Ghana and Rwanda, both of which have introduced schemes with effective government control and support coupled with intensive implementation programmes. Poor support for CBHI is repeatedly linked elsewhere with failure to engage and account for the ‘real world’ needs of beneficiaries, lack of clear legislative and regulatory frameworks, inadequate financial support, and unrealistic enrolment requirements. Nigeria’s CBHI-type schemes for the informal sectors of its NHIS have been set up under an appropriate legislative framework, but work is needed to eliminate regressive financing, to involve scheme members in the setting up and management of programmes, to inform and educate more effectively, to eliminate lack of confidence in the schemes, and to address inequity in provision. Targeted subsidies should also be considered.

**Conclusions:**

Disappointing uptake of CBHI-type NHIS elements in Nigeria can be addressed through closer integration of informal and formal programmes under the NHIS umbrella, with increasing involvement of beneficiaries in scheme design and management, improved communication and education, and targeted financial assistance.

## Background

Universal healthcare coverage (UHC) has been difficult to achieve in many developing countries, with large populations remaining over-reliant on direct (out-of-pocket, OOP) expenses that include over-the-counter payments for medicines and fees for consultations and procedures [1]. The World Health Organization (WHO) views medical fees as a significant obstacle to healthcare coverage and utilisation, and has stated that the only way to reduce reliance on direct payments is for governments to encourage the risk-pooling prepayment approach [1]. In this environment, community-based health insurance (CBHI) has emerged as an alternative to user fees. CBHI schemes are designed to ensure that sufficient resources are made available for members to access effective health care [2]. Contributions are accumulated and managed to spread the risk of payment for health care among all scheme members, although CBHI is known to be particularly vulnerable to adverse selection, where disproportionate enrolment by high risk contributors accompanies non-participation by low risk individuals [2,3].

There are various forms of CBHI, including mutual health organisations, medical aid societies and micro-insurance schemes. All are voluntary and apply the basic principle of risk sharing [3]. Unfortunately, some CBHI schemes operating in sub-Saharan Africa (SSA), including that of Nigeria, have been hampered by low enrolment rates, limited resource mobilisation and poor sustainability [4].

Health care provision in Nigeria is the responsibility of the three tiers of government, with the university teaching hospitals and federal medical centres (tertiary health care) being run by the federal government, while state governments manage general hospitals, and local government focuses on dispensaries (which are regulated by the federal government through National Primary Health Care Development Agency, or NPHCDA) [5]. Although expenditure on health rose from 12.5 million Nigerian naira in 1970 to 98.2 million in 2008, the healthcare system remains inefficient [5]. After independence in 1962, the Nigerian government was able to use oil revenues and general taxation to fund universal free health care mainly via public facilities [6]. However, the global slump in oil prices during the 1980s, coupled with economic and political instability and the general poor state of Nigeria’s health services, led to the need to rebuild Nigeria’s health infrastructure [6]. In May 1999 the Nigerian government created the National Health Insurance Scheme (NHIS), with formal enabling of private sector participation to reflect Nigeria’s operation of a mixed economy and to correct the previous poor integration of private health facilities [5,6]. Although the enabling law was signed in 1999, Nigeria’s NHIS did not in fact become fully operational until 2005 [7].

The Nigerian NHIS is an agency under the Nigerian Federal Ministry of Health and consists of three main programmes: one formal and two informal [6,8,9]. The Formal Sector Social Health Insurance Programme (FSSHIP) covers public employees and the organised private sector, and is implemented via a managed care model funded through percentage contributions from employers and employees. It is mandatory for organisations with ten or more employees [6,8,9]. The two other schemes, the Urban Self-Employed Social Health Insurance Programme (USSHIP) and The Rural Community Social Health Insurance Programme (RCSHIP) are outside the formal sector and are non-profit voluntary schemes based on the CBHI model. Revenue is generated for the USSHIP by flat-rate monthly payments with contributions dependent on the health package chosen, whereas RCSHIP members acquire accreditation according to their health needs and then choose benefits, with cash contributions being made as flat-rate monthly payments or in instalments.

Financing is not linked to ability to pay for either the USSHIP or RCSHIP, both require high levels of self-management, and benefit packages are not fully comprehensive [6,9]. Enrolment has been disappointing, with around 5 million people in all three NHIS programmes (~3% of the population) [7,8]. Nevertheless, the Executive Secretary of the NHIS, Dr Martins Olufemi Thomas, has recently assured Nigerians of his determination to attain the president’s target of 30% coverage by 2015 [10]. The need for equitable health care that responds to the needs of the people of Nigeria has been highlighted recently by a cross-sectional study of the semi-urban Samaru community that showed over a quarter of families having difficulty settling their medical bills [11]. Catastrophic health expenditure was shown by the need to sell assets or borrow money, or even to resort to begging.

Equity of healthcare access in Nigeria has been discussed recently [6], and the present review therefore focuses on ways in which participation in the CBHI elements of the Nigerian healthcare system might be improved. The aim of this study is to provide an overview of the present status of CBHI in Nigeria and other countries in SSA to identify and summarise the issues that affect the successful implementation and sustainability of these programmes. The experience of CBHI in other countries will then be drawn upon to make recommendations for improving the uptake and sustainability of CBHI in the informal sector in Nigeria.

## Methods

### Literature searches

Searches of PubMed and EconLit were undertaken using the search string (Community-based health insurance OR Community health insurance) AND (Nigeria OR Ghana OR developing countries OR Senegal OR Rwanda OR Cameroon OR Africa OR Uganda OR Kenya OR Tanzania OR Guinea) NOT India. Criteria for inclusion were that papers should report analyses of CBHI uptake and policy implications that were informative in identifying barriers to enrolment and sustainability. Meta-analyses and authoritative reviews on the subject were also included. Other relevant sources that were utilised for key demographic, health and economic data included principally websites of organisations such as the World Health Organization (WHO), official government NHIS websites of countries of interest, the World Bank and the Center for Health Market Innovations.

Although papers detailing research in Asian countries were not included in the main analysis, points of interest from the Asian experience of CBHI that became apparent during the literature review are briefly covered in the discussion to increase the generalisability of the findings.

### Analysis of data

Papers were selected with the assistance of an independent expert (JN). We accepted literature that dealt specifically with CBHI schemes based on the model of voluntary participation and the concept of risk pooling, and that set out to analyse factors contributing to recruitment and retention. We also included relevant reviews and discussion papers, and official or policy documents that dealt with reasons for and potential solutions to difficulties with CBHI enrolment. To aid with selection, papers were tabulated in a spreadsheet and ordered by country and classified as studies, reviews or other. After sorting and obtaining full copies of the papers selected, we examined each publication for information on factors relevant to the success or otherwise of CBHI schemes, with particular emphasis on observations recurring across different countries. Positive and negative contributors to successful CBHI implementation that had been identified were then tabulated with a listing of countries where these factors had been noted. The observations were used to formulate recommendations for policy decisions with the potential to improve uptake in Nigeria and other countries where progress with CBHI has been poor.

## Results

The search yielded 65 references, of which 26 were selected for inclusion (Table 1). The large majority of papers were based on cross-sectional and/or household surveys, case studies and interviews. Two studies [12,13] were carried out from a provider perspective and highlighted problems with poor understanding of schemes by ministry staff and conflicts between subscriber and promoter values. Several studies focused on willingness to pay [14-17]. There were two reviews, one descriptive [18], which discussed policy initiatives to improve uptake, and one systematic [19], which showed CBHI to be a primary factor driving maternity services, both from Rwanda. Other data were sourced from official sources such as the Nigerian government’s NHIS website [9] and the WHO [20], and from a review of the situation pertaining to Nigeria by a key opinion leader [9]. A further study was identified from the reference list of a key WHO report that was used as a comprehensive source of background information [20].

**Table 1:** Literature search findings

**Reference**	**Type of paper**		**Main findings pertinent to the present analysis**
**Nigeria**			
Adinma, 2010 [21]	Descriptive cross-sectional study with interventional component carried out in 120 women of reproductive age at Obionu Health Centre, Igboukwu		Benefit shown for integration of maternal health services into CBHI schemes
Dienye, 2011 [22]	Questionnaire cross-sectional survey of 229 surgical patients who consented to the study		Patients paid for care mostly with personal savings; most were unaware of the NHIS. When informed, 84.3% were willing to enrol. Information must be disseminated to promote acceptance of CBHI in rural areas
Onwujekwe, 2010 [23]	Questionnaire survey of 3070 households selected by simple random sampling. Contingent valuation was used to determine WTP using the bidding game format. Correlations between socioeconomic status and geographic locations with WTP were investigated. Log-ordinary least squares was used to examine the construct validity of elicited WTP		Economic status and place of residence influence WTP for CBHI membership. Consumer awareness should be promoted, and government or donor subsidies are needed to ensure success and sustainability
Onwujekwe, 2010 [24]	Questionnaire survey of 3070 households selected by simple random sampling. Focus group discussions were used to collect qualitative data, which was then examined for links between benefit package preferences with socioeconomic status and geographic residence of the respondents		Rural and poorer households preferred comprehensive packages; urban dwellers and the better off preferred more basic packages. Long-term viability must be promoted by quick access to care and benefits, and reduction in cost of treatment
Onwujekwe, 2009 [25]	Questionnaire survey of 971 respondents in two communities selected by simple random sampling. Data analysis examined socioeconomic status, differences in enrolment levels, utilisation, willingness to renew registration, and payments		Highlighted the need for subsidies to ensure enrolment and equitable risk protection among the very poor
Onwujekwe, 2011 [26]	Questionnaire survey of 3070 randomly selected households. Head of household or most senior member interviewed, and acceptability of CBHI scored on a scale of 1 to 10		Greatest willingness to enrol detected among the poorest households. Less poor groups may be more aware of shortcomings in programmes, and may therefore be more likely to express distrust and cynicism about the success of the scheme
**Senegal, Mali & Ghana**			
Jütting, 2003 [27]	Senegal	Survey of 346 randomly selected households (2860 persons): 70% members and 30% non-members. Models used to examine impact of CBHI on health care use and expenditure	Members more likely to use facilities than non-members, and pay substantially less when they do. The very poorest households do not enrol, however: cost of participation must be reduced by lowering of prices or addition of subsidies
Ouimet, 2007 [12]	Senegal	Study of all Senegal CBHI providers, including interviews with subscribers and promoters, logistical analysis of links between subscribers and organisations and composite indicators representing values	Showed conflicts between promoter and subscriber values
Smith, 2008 [28]	Senegal; Mali; Ghana	Data from three household surveys carried out by USAID-funded Partners for Health Reform*plus*. After presentation of descriptive statistics, multiple regression was used to estimate relationships between CBHI membership and access to formal maternal health services	CBHI membership is positively associated with maternal health service use. CBHI is a potential demand-side mechanism to increase maternal health care access, but complementary supply-side interventions to improve quality of and geographic access to care are also critical
**Rwanda**			
Bucagu, 2012 [19]	Rwanda	Systematic review of literature, national policy documents and three Demographic & Health Surveys (2000, 2005 and 2010) to identify health system factors driving maternity service improvements	CBHI identified as a primary factor, together with better leadership and governance
Schmidt, 2006 [29]	Rwanda	Analysis of data from six household surveys	The goals of maximising health revenue and maximising participation in CBHI are mutually exclusive. The top three quartiles of the Rwandan population were able to contribute US$1 per capita per year, but subsidies were recommended to extend coverage to the poorest quartile
Dhillon, 2012 [30]	Rwanda	Investigation of the impact of subsidising CBHI enrolment, removing point-of-service co-payments, and improving service delivery on health facility utilisation rates in Mayange, a rural area containing approximately 25,000 people	Improvement of service delivery and reduced financial barriers (elimination of copayments and increased subsidies) increases health facility utilisation under CBHI
Schneider, 2006 [31]	Rwanda	Cross-sectional household survey data collected in 2000 in the context of the introduction of CBHI: 3139 households (354 insured and 2785 uninsured) - 14,574 individuals in total. Analysis via an indirect standardisation approach used to measure health inequality	Substantial inequality in utilisation linked to user fees - these were linked to horizontal inequity in service use across scheme members. In addition, benefit packages need to be large enough to protect households against catastrophic expenditure
Logie, 2008 [18]	Rwanda	Descriptive review summarising three health system developments introduced by the Rwandan government to lower barriers to care: coordination of donors and external aid with government policy, and monitoring the effectiveness of aid; a country-wide independent CBHI scheme; and the introduction of a performance-based pay initiative	Annual fee too expensive for the very poor and insufficient to fund basic care - extra central funding and donor contributions needed. Addition of contributions from other insurance schemes and exemptions for the poor recommended
**Uganda**			
Basaza, 2010 [13]	Uganda	Semi-structured interviews with senior Ministry of Health staff and District Health Officers - qualitative study	Revealed gaps in knowledge and understanding of schemes among Ministry of Health and District Health Office staff. Also highlighted OOP expenditure as a problem
Basaza, 2007 [32]	Uganda	Case study of two CBHI schemes: review of scheme records, key informant interviews and exit polls with both insured and non-insured patients	Various demand and supply side factors identified
Basaza, 2008 [33]	Uganda	Reasons for low enrolment were investigated in two different models of CBHI. Focus group discussions and in-depth interviews were carried out with members and non-members to acquire more insight and understanding in people’s perception of CHI, in reasons for joining/not joining and in the possibilities for increased enrolment.	Highlighted scheme design problems, ability to pay premiums, poor quality of care, trust, etc.
Kyomugisha, 2009 [34]	Uganda	Qualitative descriptive cross-sectional study: focus group discussions with scheme members and non-members (158 participants)	Schemes were not sustainable because of small budgets, low enrolment and lack of government support. Effect of abolition of user fees on scheme enrolment was minimal. Governments should ensure that quality does not suffer when user fees are removed, and schemes need substantial support to build sustainability
**Burkina Faso**			
De Allegri, 2006 [35]	Burkina Faso	In-depth interviews with 32 heads of households in Nouna District, BF	Previously neglected factors, such as institutional rigidities and socio-cultural practices, are important in shaping the decision to enrol
Dong, 2003 [15]	Burkina Faso	WTP study: household survey involving 2414 individuals and 705 household heads. Take-it-or-leave-it (TIOLI) and bidding game methods used to determine WTP	Pointed out the importance of considering differences between the theoretical and real markets, and between WTP and the costs of benefit packages
Dong, 2004 [16]	Burkina Faso	WTP survey: random sample of 698 household heads interviewed with bidding game method.	Decision makers need to consider WTP when setting enrolment units and premiums
Dong, 2004 [17]	Burkina Faso	Focus group discussions carried out after a pilot based on three key informant interviews; followed by a household survey (160 households). Qualitative survey with costings; the bidding game method was used to determine WTP and feasibility of running CBHI+	Subsidies highlighted; household characteristics influenced preferences
Dong, 2009 [36]	Burkina Faso	Survey of 756 rural and 553 urban households. logistic regression was used to study the influence of individual and household factors on CBHI drop-outs	Drop-out rates influenced by affordability, health needs and health demand, quality of care, and household head and household characteristics
Parmar, 2012 [37]	Burkina Faso	4-year study of adverse selection and targeted subsidies. CBHI was randomly offered to 41 villages and 1 town (Nouna) during 2004–6, with premium subsidies offered to poor households in 2007. Data were subsequently collected by household panel survey from randomly selected households (n = 6795); fixed effect models were applied	Targeted subsidies may increase coverage but may also increase adverse selection. Such subsidies for the very poor or other high-risk groups must be accompanied by strategies to bridge the financial gap created by adverse selection and thus assist sustainability
Souares, 2010 [38]	Burkina Faso	Community wealth ranking was used to identify the poorest quintile of households among 7762 in Nouna district who were then offered insurance at half the usual premium for 2007	Annual enrolment increased from 18 households (1.1%) in 2006 to 186 (11.1%) in 2007
**Cameroon**			
Donfouet, 2011 [14]	Cameroon	Contingent valuation study based on survey of 410 rural households. Willingness to pay investigated	Substantial demand for CBHI in rural Cameroon, but social marketing strategies such as mass media campaigns are needed to raise awareness
**Guinea**			
Criel, 2003 [39]	Guinea	Focus group discussions carried out to explore reasons for low enrolment	Poor quality of care highlighted as a factor

CBHI, community-based health insurance; NHIS, National Health Insurance Scheme; WTP, willingness to pay.

The results of the review are reported in Table 2 under four columns that capture both positive and negative influences regarding CBHI and social health insurance (SHI) uptake: factors, example countries for each factor, issues identified and policy implications.

**Table 2:** Positive and negative factors influencing uptake of CBHI and other forms of social health insurance in SSA, and implications for policymakers

**Factor**	**Examples of countries where factors noted**^**a**^	**Issues identified and policy implications**	**References**
**Factors positively linked to uptake**			
Provision of uniform benefit packages offering wide illness coverage	Ghana	Benefits should be predefined and comprehensive, with a good coverage of likely disease burden	NHIS [Ghana] [9]; Odeyemi & Nixon [6]
		Provision of services at accredited facilities helps to ensure uniformity of benefits offered	
Adequate public financing/realistic pricing	Ghana, Rwanda, Burkina Faso	Use of funds from taxation is necessary to allow funding to become progressive and to encourage/enable the less well-off to join through subsidies and fee exemptions	NHIS [Ghana] [40]; Odeyemi & Nixon [6]; Logie [18]; Schmidt [29]; De Allegri [35]; Parmar [37]; Souares [38]
		Targeted subsidies positively influenced enrolment in Nouna, BF, although there is also a danger of adverse selection	
Elimination or minimisation of copayments	Rwanda	Increases in subsidies to the point where copayments are eliminated could lead to as much as 100% coverage	Dhillon [30]; Schneider & Hanson [31]
		User fees in Rwanda were found to be linked to substantial inequality in utilisation, with medical visits being more common among the more well-off uninsured	
Strong desire/willingness to join	Cameroon, **Nigeria**	Greatest willingness noted among poorest households in Nigeria	Donfouet [14]; Onwujekwe [26]
		Policy makers should undertake research to determine WTP; social marketing can encourage participation	
Avoidance of focus on maximisation of health revenue	Rwanda	CBHI participation and a focus on the generation of healthcare revenues are mutually exclusive	Schmidt [29]
Improvements in education and socioeconomic status	Burkina Faso	Enrolment in schemes may increase in line with social and economic progress and development over the long term	De Allegri [35]
Provision of maternal healthcare benefits	Senegal, Mali, Ghana, Rwanda, **Nigeria**	Inclusion of maternal healthcare benefits promotes interest in CBHI as a demand-side driver, and CBHI is a primary contributor to strong maternal health services	Smith [28]; Bucagu [19]; Adinma [21]
		Scheme organisers should ensure that packages are comprehensive, as excessive limitation discourages uptake	
Awareness of the limitations of traditional medicine	Burkina Faso	Noted in the Nouna, BF survey. Further research is needed, but this observation emphases the value of improved education and communication	De Allegri [35]
**Negative factors that discourage or limit uptake**			
Excessive requirement for OOP expenditure, inability to pay	Uganda, Burkina Faso, Guinea, Senegal, **Nigeria**	Major determinant of enrolment; even where implementation has been predominantly successful, the very poorest populations may still find participation financially difficult	Basaza [13,32,33]; Dong [36]; Criel [39]; Onwujekwe [25]; Metiboba [7]; Jütting [27]
		OOP remains significant in healthcare systems in many countries (despite actions such as abolition of user fees in government institutions in Uganda)	
		Regressive flat-rate payments are a problem in Nigeria, and inability to pay premiums is the single biggest obstacle in Uganda. There are no mechanisms in place to help those who cannot afford to join	
		Ambiguous and contradictory healthcare funding policy is a significant problem that must be addressed	
Social exclusion due to religion or ethnicity	Senegal	Noted in Senegal, where the Roman Catholic Church supports the *Mutuelles*, and where Christians were reported in 2003 to be more likely than Moslems to enrol. In interviews, Moslems were under the mistaken impression that CBHI was open to Christians only	Jütting [27]
Lack of legal framework or umbrella organisation	Guinea, Benin	Failure to provide any proper governance or official framework for CBHI schemes is linked to low enrolment	Soors [20]
Lack of government (or donor) support	Uganda, Burkina Faso, **Nigeria**	Small budgets, low enrolment and lack of government support cause schemes to fail. Schemes need substantial support to build their sustainability; technical and policy decisions should account for this	Kyomugisha [34]; De Allegri [35]
Excessively rigid enrolment requirements or institutional rigidity	Uganda, Burkina Faso	Failure to recruit the required number of people in a village has been a key feature affecting schemes in Uganda (mandatory 60% of a group or 100 families per village)	Basaza [32,33]; De Allegri [35]; Onwujekwe [25]; Onwujekwe [23]
		Rules for group membership should reflect what is achievable	
Mismatch of values expressed by promoters and subscribers; failure to align the ‘real’ market with the theoretical one, and to match benefits with WTP	Senegal, Burkina Faso, **Nigeria**	Need to align expectations/needs of promoters (focus on financial sustainability) and subscribers (who look for sustainability and solidarity)	Ouimet [12]; Dong [15-17]; Onwujekwe [23]; Onwujekwe [24]; Metiboba [7]
		Increase participation of members in decision making; failure to engage beneficiary participation in Nigeria has been pinpointed as a major problem	
		Ensure that prospective members are willing to pay for the benefits on offer, and that the market in any locality matches the theoretical one on which projections are based	
Lack of information	Uganda, Burkina Faso, **Nigeria**	Governments and promoters must ensure that schemes are properly and accurately publicised, and the public properly informed; lack of knowledge can lead to scepticism	Basaza [13]; De Allegri [35]; Dienye [22]; Onwujekwe [23]; Metiboba [7]
		Lack of information is a significant problem in Nigeria	
		Authorities must ensure that government and health officials are fully informed about the packages on offer	
Poor quality of healthcare	Uganda, Guinea	Concerns relate to cleanliness, long queues before being seen, and lack of some prescribed medicines	Basaza [32,33]; Criel [39]
		Noted as the main reason for lack of interest in the Maliando Mutual Health Organisation in Conakry, Guinea	
Lack of trust; perception that schemes are unfair or even unnecessary; dislike of health care personnel and cultural resistance	Uganda	Belief that non-members are treated better in hospital than scheme members	Basaza [32,33]; Kyomugisha [34]
		Integrity of fund managers and transparency of operation: “Nothing is done to ensure that fund managers account to scheme members” (Ugandan interview respondent)	
		Some members pay premiums continuously but never fall sick	
		“I wasn’t bothered since I am young and not likely to fall sick”; “If I do not fall sick, I should not pay for someone else” (Ugandan survey respondents)	
		Schemes must be fair, well run and affordable, and the public sufficiently well-informed to appreciate the need for coverage and mutuality	
High drop-out rates	Burkina Faso	Related to other factors noted in this table: affordability, health needs and demand, quality of care and household characteristics	Dong [36]
		Improve perception of schemes by heads of households, ensure that large households are able to maintain contributions (e.g. flexibility in payment options); ensure that service offered meets expectations (e.g. in line with education, etc.)	

a. Listings of countries are not intended to be comprehensive but indicate locations where the issues raised have been identified as significant factors. **Nigeria** is shown where studies have highlighted a particular topical issue for that country.

CBHI, community-based health insurance; OOP, out-of-pocket; SSA, sub-Saharan Africa; WTP, willingness to pay.

### CBHI schemes and their uptake in Nigeria and other SSA countries

#### ***Nigeria***

It is difficult to find up-to-date details of schemes currently running in Nigeria or recent national estimates of participation rates. Moreover, as mentioned earlier, the NHIS was fully implemented only relatively recently (2005). Metiboba reported in 2011 a statement made in 2009 by Audu, the executive secretary for the NHIS, that only 3% of the entire Nigerian population was covered [7]. Underlying problems have been reviewed more recently by Baba & Omotara [5], who place the poor performance of Nigeria’s NHIS within the wider context of a fragmented approach to healthcare that involves both federal and state governments, a deterioration in the public health service caused by a ‘brain drain’ and lack of resources, and the high levels of poverty encountered in Nigeria [5].

Examples of implementation of CBHI under Nigeria’s mixed informal sector model include the Hygeia Community Health Plan (HCHP), the first programme to be run in Lagos and Kwara State under the oversight of the Health Insurance Fund (HIF), an international not-for-profit organisation that undertakes to deliver private health insurance and services to low and middle income families. An ongoing study is assessing the ability of the HCHP to provide cardiovascular disease preventive care in a low resource setting [41].

Other studies have focused on communities in Anambra State, where a government/community healthcare co-financing scheme is in operation [21,25]. This scheme mobilises the community via a health committee which has overseen refurbishment and re-equipping of the publicly owned health centre, sourced drugs and employed or re-deployed health personnel. The community members themselves set the premium to enrol at 100 and 50 Nigerian naira per month for each adult and child, respectively. The scheme focuses on maternal and child health. Enrolment in the scheme was reported to be 15.5% in a non-successful community but 48.4% in a successful one [25]. High enrolment was linked chiefly to increased awareness of the scheme, while low enrolment was linked to regressive contributions, failure to fit implementation to a specific area, insufficient community involvement, and lack of trust in the scheme and its management [25].

#### ***Senegal, Mali and Ghana***

Gains in Senegal surpass those in most other African countries (17.9% participation in non-formal CBHI by 2007), particularly in reduction of OOP payments, but organisational difficulties prevail: a legal framework for CBHI was established in 2003 but was still under revision in 2010 [20]. Participation in Mali remains very poor, with OOP expenditure rising between 2004 and 2007 [20]. The Malian government is attempting to address this by merging three existing schemes (formal sector coverage, medical assistance for the indigenous population, and *mutuelle* health organisations for the informal sector) and attempting to extend health coverage through the *mutuelles*.

In contrast, Ghana’s NHIS scheme has been relatively successful [6,40]: 66.4% of the population had been covered by June 2010, with 29.6% in the informal adult sector. The Ghanaian NHIS is of special interest because of the way it adapts the SHI model to include informal workers. The combination of a network of CBHI-type entities (the District-Wide Mutual Health Insurance schemes) with a centralised authority and source of funding (the National Health Insurance Fund) ensures potential nationwide coverage and helps to guarantee financial sustainability. Thus, the NHIS adapts the best aspects of two very different health financing models to fit Ghana’s specific socioeconomic requirements [42].

#### ***Rwanda***

Rwanda’s health system is financed both by state funds and by individual contributions through health insurance and direct fees for services. Civil servants, pensioners who have previously contributed towards medical care and certain private institutions are covered by a medical insurance scheme overseen by the Rwanda Social Security Board [43]. To achieve universal coverage, the government of Rwanda has established a social health insurance programme called the *Mutuelles de Santé.* The scheme has been in place since 1999 and has been highly successful, with CBHI covering the entire country by 2005 [20]. An evaluation of the scheme’s performance to 2008 showed improved medical care utilisation and protection of households from catastrophic health spending under CBHI, with Rwanda being the only country in SSA to achieve >90% coverage [44]. Benefits are provided at two levels: a Minimum Package of Activities to cover all services and drugs at health centres, and a Complementary Package of Activities covering limited hospital services. Premiums and copayments vary, but the government covers these costs for the indigent population and other vulnerable groups [45]. The key to Rwanda’s success in implementing CBHI appears to lie in a strong societal consensus over equality of access to health care with financial protection, crucially supported by the government through investment in the health sector, effective legislation to provide basic care for the uninsured, and an intensive nationwide programme [44].

A report from Rwanda in 2006 indicated significantly higher utilisation rates for both actual and needs-adjusted medical visits among scheme members than among persons paying user fees [31].

#### ***East African Countries (Uganda, Tanzania, Kenya)***

These countries are characterised by very poor progress and have been undermined by serious challenges from high levels of poverty, population growth, a very high prevalence of HIV/AIDS and chronic underfunding of the health sector. Very poor participation is typical [46]. Most information comes from Uganda, where CBHI operates in the ~30% of the system covered by the private not-for-profit sector: participation in Uganda suffered badly in 2001 when user fees were abolished in the public system (~60% of facilities) [13,20]. In 2007, only 2% of Ugandans eligible for CBHI were actually enrolled [32].

#### ***Burkina Faso***

Enrolment remains marginal and OOP expenditure is very high in Burkina Faso. Research focuses on a pilot CBHI scheme that was implemented in the Nouna Health District in 2004 [17,38,47]. As poverty has been identified as a key barrier to enrolment, the authorities have used community wealth ranking to identify the poorest households, who then receive significant subsidies. This approach has been found to be effective and has been recommended for use elsewhere [38].

#### ***Cameroon***

There is strong demand in Cameroon for CBHI for poor rural households [14]. This demand, however, has not been matched by increased protection from catastrophic health expenditure: in 2007, OOP expenditure remained at 94.5%. Pilot schemes have been launched and there is a stated aim to have one CBHI scheme per district by 2015, but there remains no national umbrella organisation and finance is lacking [20].

#### ***Other SSA Countries***

In Guinea, the *Union des Mutuelles de Santé de Guinée Forestière* (UMSGF) was established as part of a health insurance program in 1999, but fewer than 1.5% of Guineans had CBHI coverage in 2006, and OOP expenditure remained at 99.5% [20]. There is no legal framework or umbrella organisation for CBHI. The situation is similar in Benin (no legal framework; CBHI coverage <1.4% in 2006) [20]. Niger is encumbered by very slow progress, extreme poverty and low government capacity: schemes have been introduced but coverage remains minimal [20]. Tanzania is making modest progress with CBHI, but problems include flat-rate premiums with inability to pay and the persistence of potentially catastrophic OOP costs for referral care [20]. Data from the Democratic Republic of Congo are limited chiefly to the Bwamanda Insurance Scheme, a *mutuelle* that is achieving high rates of coverage by using latent capacity in a hospital to supply low-cost insurance to rural poor who would otherwise high pay high OOP fees [20,48].

### Drivers of success and sustainability

Factors driving the success or failure of CBHI schemes appear as recurring themes in Nigeria and other SSA countries (Table 2). Problems are chiefly operational in nature and include failure to account for the inability of the target population to pay for scheme membership, lack of clear legislative and regulatory frameworks coupled with inadequate financial support, and unrealistic enrolment requirements. The last is linked to failure on the part of administrators to align ‘real-world’ socioeconomic conditions with those suggested by theoretical models, to account for the target population’s willingness to pay for a scheme, and to engage communities in decision making from the earliest stages. Failure to engage beneficiary participation in CBHI, lack of comprehensive cover and failure to appoint an omnibus regulator have all been criticised [7]. Moreover, patient preferences may not be receiving sufficient attention, and packages suitable for one community may not suit another [24]. These issues recur across countries that have faced significant difficulties in implementing CBHI. Problems with quality of service and a lack of trust in the integrity of the schemes and their administrators and providers have also been identified, particularly in Uganda, Guinea and Burkina Faso (Table 2). Lack of awareness also continues to present barriers to enrolment [7,23]: 97% of a sample of rural Nigerian surgical patients in one survey had no knowledge of their government’s insurance Schemes [22]. Most had paid for their treatment from personal savings or had relied on family members. In Senegal, Moslems were found in the early 2000s to be much less likely than Christians to enrol because they mistakenly believed that the schemes were open to Christians only [27].

Conversely, success factors are also evident. Researchers in Cameroon and Nigeria have documented a clear desire on the part of less well-off households to join schemes (Table 2), and over 3000 Nigerian survey respondents stated that CBHI was an acceptable means of paying for health regardless of socioeconomic background or location [26]. Interestingly, the poorest households expressed the greatest willingness to enrol. Countries such as Ghana and Rwanda have shown key drivers of success to include uniform and comprehensive benefit packages, adequate finance by government and elimination or minimisation of copayments. Flat-rate payments are likely to discourage the very poor [9,25], and the use of funds from taxation has been shown to be necessary to avoid the need for regressive member contributions. Even in a relatively successful country such as Senegal, there has been difficulty in reaching the very poorest members of society, which has highlighted the need for subsidies [27]. Targeted subsidies for the most disadvantaged have a positive influence as long as adverse selection is avoided [21,23] (Table 2).

Providing a specific focus for CBHI (e.g. maternity services [21]) has been shown to assist adequate funding and improve coverage in rural communities. Other initiatives of this kind include the Hygeia Community Health Plan (Lagos and Kwara State, Nigeria) that aims to improve access to cardiovascular care in low-resource settings [41].

## Discussion

In contrast to industrialised nations, which generally have fully developed publicly funded health systems and/or properly regulated insurance provision, healthcare provision in many developing countries remains fragmented and non-universal. CBHI has emerged as a potential strategy to address this. The contribution that CBHI can make to the achievement of UHC is acknowledged by international organisations such as the WHO, the World Bank and the United Nations Children’s Fund (UNICEF), despite its known shortcomings [49].

The evidence reviewed points to CBHI’s potential role in achievement of outcomes linked to the UN’s Millennium Development Goals (MDGs). For example, Goal 5 is to improve maternal health, and Adinma et al [21]. showed improved delivery and utilisation of maternal health services after the introduction of a CBHI-type scheme in Anambra State, Nigeria (see above). Similarly, in Rwanda, marked increases among rural populations in proportions of births with skilled support, deliveries in institutions, and use of contraception were reported after performance-based financing, leadership and governance within the context of scaling up CBHI programmes [19]. CBHI is also known to provide financial protection by reducing OOP spending; it increases cost recovery, financial protection and utilisation of inpatient and outpatient services [50,51]. There may also be a positive effect on social inclusion [51].

The question remains, however, as to how to expand cover effectively to those in greatest need in a way that is viable, fair and sustainable, and that delivers a high standard of service. Evidence from many countries illustrates that enrolment into CBHI schemes is driven by a variety of inter-related factors, and that poor uptake cannot simply be attributed to income or socioeconomic and geographical differences.

The evidence reviewed generally shows that most of the successful schemes are regulated properly and supported financially by central governments. Experience of CBHI in regions outside SSA supports this [49]. Most CBHI schemes in India, for example, are driven by non-governmental organisations (NGOs), with much organisational diversity [20]. Critical issues still to be addressed include flaws in design, such as minimal exclusions to reduce OOP payments, and enrolment of whole family units to control adverse selection [52]. Strategic purchasing and effective monitoring are also necessary, and financial viability could be increased through the amalgamation of smaller schemes. Most notably, the government of India has created a more supportive policy environment by granting legal recognition to CBHI schemes and providing subsidies for the poor. In order to move forward, premiums should be affordable, benefit packages comprehensive, providers regulated, and reimbursements cashless and straightforward [52].

The Indian government is taking steps to address these problems through the introduction of federally regulated schemes such as National Rural Health Mission (NRHM) of the Ministry of Health and Family Welfare, the Rashtriya Swasthya Bima Yojana (RSBY) of the Ministry of Labour and Employment, and the Rajiv Aarogyasri scheme launched by the state government of Andhra Pradesh [49]. These schemes have achieved early success, and reflect a pattern in Asian countries of moving away from informal coverage and bringing SHI schemes into regulated governmental frameworks. In Indonesia, Jamkesmas, a government-financed program for the poor, has become the largest insurance programme, and is expected to be integrated with all other SHI schemes by 2014 [49]. As part of its effort to achieve UHC, the Philippines introduced the government-financed health coverage program (HCP) for poor households in 1996. A sound design feature of the HCP was its incorporation into the National Health Insurance Program, but problems have arisen as a result of a reliance on local government units to enrol and pay for the poor, use of finance from uncertain sources (e.g. the Philippines Charity Sweepstakes), suboptimal benefit package design and accountability issues. Thus, although the health insurance landscape in Asia differs somewhat from that in SSA, there are some common patterns and lessons that can be drawn.

In Nigeria, although the USSHIP and RCSHIP offer promising ways of scaling up NHIS participation outside the compulsory formal sector, engagement with the NHIS remains primarily through the FSSHIP. There is nevertheless a strong demand for cover among the least well-off, and positive elements are already in place. These include the accreditation of providers and the requirement to include at least 500 members grouped around common economic activities (USSHIP) or a community (RCSHIP). This minimum size and requirement for coherency across the group, together with membership accreditation, would be expected to improve sustainability. The findings of the present study suggest that the uptake and sustainability of non-statutory NHIS elements in Nigeria could be improved by the following:

• Closer integration of CBHI with the formal sector under the existing NHIS umbrella, with improved regulation and guarantee of financial stability by central government. Government support via tax revenues is necessary to cover gaps and ensure sustainability.

• Elimination of regressive funding (flat rate payments).

• Elimination of unrealistic pricing and minimisation of OOP payments, equity of availability of benefit packages, and consideration of targeted public subsidies.

• Improved understanding of beneficiary needs by policymakers. Scheme benefits need to be comprehensive and easily understood, and administrators and providers trusted by beneficiaries. Evidence strongly suggests that current lack of confidence is being driven by lack of awareness and poor communication, which could be addressed through targeted information campaigns.

• Increased citizen participation in the organisation and running of schemes (the Nigerian model provides for member involvement). Information about the schemes available should be widely disseminated and understood.

• Ensuring transparency and monitoring of quality of service: communities may not be enrolling because of lack of information or misinformation, lack of trust and cultural resistance, or a perception that schemes are unfair and the standard of care is inadequate.

• Monitoring of risk sharing, claims-to-revenue ratios and operating costs to ensure long-term sustainability [51].

Closer scheme integration and increased government support would reflect the situation in Ghana and Rwanda, where voluntary programmes have been more successful. Similar thinking can be found elsewhere in SSA, as exemplified by the comments of a senior Ugandan ministry official in one survey: “Health is something that everyone needs to maintain, and therefore community health insurance has a place in Uganda. Let us start with national policies facilitating community health insurance. Regulations are very important and gradual implementation is needed” [13].

Attempts to cover wide gaps with public funding may prove too great a burden for the general taxation system, in which case donor funding may be an option. This is being pursued in Nigeria through grants from the International Finance Corporation (IFC), but health care as a proportion of total spending was only 4.9% in 2009, so there is a great deal of scope for increased national spending on health to meet gaps in healthcare provision [6].

CBHI is by its nature voluntary, and the WHO has expressed doubts about the efficacy of voluntary participation in the drive to achieve UHC [1]. As described earlier in this review, voluntary schemes are vulnerable to adverse risk selection which can lead to inadequate risk pooling [3]. Conversely, however, voluntary schemes can raise funds in the absence of widespread prepayment and pooling. They can also familiarise people with the benefits of insurance and prepayment, although they have a limited ability to cover a range of services for those too poor to pay premiums [1].

Introduction of compulsion at some level might be controversial and difficult to implement, especially in the informal sector and in remote settings, and conflicts with the voluntary CBHI model. In contrast, SHI schemes elsewhere are often statutory with safety nets being provided through general taxation. A major challenge for Nigeria is poverty in rural areas, and there is little room for imposition of penalties. However, this study demonstrates many lessons from a variety of countries and initiatives that could make CBHI an effective tool in widening health care.

In terms of limitations, it is recognised that this study is based on a search of two electronic databases (PubMed and EconLit) and relevant websites, and informally published ‘grey’ literature was not examined in detail. The systematic approach used, however, is likely to have captured relevant studies and also provides an exemplar for future research relevant to the successful implementation of CBHI programmes in developing countries.

## Conclusions

Implementation of CBHI-type programmes within the NHIS of Nigeria has been disappointing to date, but experience elsewhere suggests that uptake and sustainability could be improved through policies that include closer integration of the informal and formal sectors under the existing NHIS umbrella with increasing involvement of beneficiaries in scheme design and management, improvements in communication and education, higher public and private healthcare funding, and targeted financial assistance. These findings are likely to be instructive for policymakers in SSA generally in achieving UHC goals and improving health outcomes.

## Competing interests

Isaac A.O. Odeyemi is an employee of Astellas Pharma UK Ltd.
